# A *WAS* promoter variant underlying Wiskott-Aldrich syndrome in two kindreds

**DOI:** 10.70962/jhi.20250181

**Published:** 2025-10-30

**Authors:** Pauline Ober, Christelle Lenoir, Arnaud Maillard, Marie-Gabrielle Vigue, Marjolaine Willems, Sandrine Baron-Joly, Carole E. Aubert, Eva Maria Tinner, Nathalie Lambert, Iben El Missaoui, Frédéric Parisot, Antoine Fayand, Yoann Seeleuthner, Sylvain Hanein, Tom Le Voyer, Martin Broly, Guilaine Boursier, Jean-Laurent Casanova, Peng Zhang, Jana Pachlopnik Schmid, Sylvain Latour, Jérémie Rosain

**Affiliations:** 1 Imagine Institute, Inserm U1163, University of Paris Cité, Paris, France, EU; 2 Laboratory of Human Genetics of Infectious Diseases, Necker Branch, Inserm U1163, Necker Hospital for Sick Children, Paris, France, EU; 3 Laboratory of Lymphocyte Activation and Susceptibility to EBV Infection, Inserm U1163, Imagine Institute, Paris, France, EU; 4 Pediatric Immunology and Infectiology Unit, University Hospital of Montpellier, Montpellier, France, EU; 5 Department of Medical Genetics, University Hospital of Montpellier, Montpellier, France, EU; 6 Department of Pediatrics, University Hospital of Nîmes, Nîmes, France, EU; 7 Department of General Internal Medicine, Inselspital, Bern University Hospital, University of Bern, Bern, Switzerland; 8 Institute of Primary Health Care (BIHAM), University of Bern, Bern, Switzerland; 9 Paediatric Haematology/Oncology, Department of Paediatrics, Inselspital, Bern University Hospital, University of Bern, Switzerland; 10 Study Center for Primary Immunodeficiencies, Necker Hospital for Sick Children, Assistance Publique Hôpitaux de Paris (AP-HP), Paris, France, EU; 11 Bioinformatic Platform, Institute of Genetic Diseases, Inserm U1163, Imagine, University of Paris Cité and Structure Fédérative de Recherche Necker, Paris, France, EU; 12 St.Giles Laboratory of Human Genetics of Infectious Diseases, Rockefeller Branch, Rockefeller University, New York, USA; 13 Clinical Immunology Department, Assistance Publique Hôpitaux de Paris (AP-HP), Saint-Louis Hospital, Paris, France, EU; 14 Department of Molecular genetics and Cytogenomics, Autoinflammatory Diseases Unit, Montpellier University Hospital, University of Montpellier, CEREMAIA, IRMB, INSERM U1183, Montpellier, France, EU; 15 Department of Pediatrics, Necker Hospital for Sick Children, AP-HP, Paris, France, EU; 16 Howard Hughes Medical Institute, New York, NY, USA; 17 Division of Immunology, University Children’s Hospital Zurich, Zurich, Switzerland; 18 Pediatric Immunology, University of Zurich, Zurich, Switzerland

**Keywords:** Inborn error of immunity, genetics, non-coding variants, promoter

The global expansion of genomic medicine has improved access to genetic testing for patients with suspected inborn errors of immunity (IEI). Despite these advances, diagnostic rates remain relatively low, with current estimates suggesting that fewer than 50% of patients with suspected IEI receive a confirmed genetic diagnosis. This limitation is partly due to the focus of most clinical laboratories on variants within protein-coding regions or those impairing splicing^1^. Expanding variant analysis beyond these protein-coding regions and variants leading to aberrant splicing is promising to improve the diagnosis of IEI. Such variants can be pathogenic through different mechanisms, such as disruption of the 5’ untranslated region (UTR) and 3’ UTR, or by disrupting cis-regulatory elements (CREs), including promoters or enhancers^2,3^. While the availability of tools to screen for variants disrupting 5’ UTR and 3’UTR remains limited, many scores or strategies were developed recently for variants located in cis-regulatory elements (CRE), including the use of tailored cut-offs for combined annotation dependent depletion (CADD) and regulatory Mendelian mutation (REMM) scores^2^. A specific tool to screen for such variants, promoterAI, was also recently published.

In this context, we investigated four male patients from two unrelated kindreds with features of IEI (Figure A). P1 and P2, from kindred A, are of French descent and were born to non-consanguineous healthy parents. P1 is four years old, and has a history of eczema, celiac disease and recurrent hemorrhagic immune thrombocytopenic purpura (ITP) (Figure B and C). P2 is seven years old and suffered at the age of two years from an acute episode of ITP (Figure B and C). He also has a history of atopic dermatitis. P3 and P4 from kindred B were born to non-consanguineous Eritrean parents and currently reside in Switzerland. Both patients presented with eczema. P3, now 24 years old, was referred in early childhood for lymphadenopathy. At the age of five, he was diagnosed with EBV-driven Hodgkin lymphoma (mixed-cellularity subtype, EBV positive, stage IIAE) involving the cervical, supraclavicular and mediastinal regions. Almost two years after completing chemotherapy, he experienced a relapse of Hodgkin’s disease with axillary, mediastinal, intra-abdominal and pulmonary involvement (stage IVa). At nine years of age, he developed autoimmune hemolytic anemia and ITP, initially treated with steroids. P3 has since experienced multiple ITP relapses and received treatment with steroids, intravenous immunoglobulins (IVIG) and rituximab. He is currently stable under immunosuppressive therapy with mycophenolate-mofetil (MMF), prednisone, and sirolimus. The brother of P3, P4, is 32 years old and suffered from diffuse large cell lymphoma, classified as Murphy stage III (equivalent to Ann Arbor stage III) and was treated with chemotherapy. Until now, he has been in remission but has persistent inguinal lymphoproliferation, where biopsies were taken without any signs of malignancy. The sister and parents of P3 and P4 are healthy.

Given the suspicion of IEI, both kindreds were explored through high-throughput sequencing (HTS) approaches capturing exons, *e.g.* a panel for P1, and whole-exome sequencing (WES) in both P3 and P4. Screening of small nucleotide variants or copy number variants within the coding sequences and essential splice site variants of captured genes failed to identify any pathogenic variants. We thus reanalyzed for variants covered in non-coding regions that are partially covered even by exon-captured based HTS^1^. We did not detect any non-coding variants predicted to impair splicing by tools such as spliceAI. We further screened on non-coding variants with high CADD score, adapting the algorithm from Villani *et al*^2^. We ended up identifying in both kindreds a single nucleotide variant (SNV) located upstream of the *WAS* gene (hg38 X-48683790-C-T; NM_0000377.3; c.−64C>T). This variant is very rare, because it is absent from gnomAD v4.1. The CADD v1.7 score was high (=20.5). In addition, this variant was predicted as deleterious by REMM v0.4 (=0.999) and promoterAI v1.0 (=−0.746). The variant was indeed located on a conserved nucleotide as highlighted by elevated phastCons (=1) and phyloP (=3.90) scores. This variant is predicted to be located in a cis-regulatory element by ENCODE, and the c.−64C position lies within the *WAS* promoter region shown to be essential for hematopoietic-specific expression, as it contains critical transcription factor binding sites (*e.g.* FLI1, GATA family motifs and GC-boxes that bind Sp1/Sp3) (Figure D)^4^. By Sanger sequencing, this variant was confirmed to be present at a hemizygous state in P1, P2, P3 and P4, at a heterozygous state in the mothers of both kindreds and in the sister of P3 and P4, and was absent in the fathers of both kindreds (Figure E). At immunological level, P1, P2, P3, and P4 displayed T CD8 lymphopenia, with increased CD4/CD8 ratio (data not shown). In T-activated blasts of the patients, WASP expression was 22% and 10% of normal at the mRNA and protein levels, respectively (Figure G–H). Overall, according to criteria from the American college of medical genetics (ACMG), these data are further supportive of categorizing the c.−64C>T variant as pathogenic and causal for the clinical phenotype of the four patients.

We thus report a pathogenic variant located within the promoter of *WAS*. We found this rare variant in two unrelated kindreds, and evidenced decreased but not absent protein level amongst the T cells of the patients. This likely explained the clinical phenotype, characterized by intermittent periods of normal platelet counts and relatively mild but fluctuating disease manifestations such as eczema, autoimmunity, and lymphoproliferation. Although this presentation may appear mild and would historically have been considered “intermittent X-linked thrombocytopenia”, it would now be better classified as a class I WAS variant^5^, and the occurrence of malignancies in two patients highlights the potential severity of such phenotypes. Clinically, this emphasizes that WASP deficiency should also be considered in patients who do not present with the classic triad (eczema, thrombopenia, and immunodeficiency) and who may even have episodes of normal thrombocyte counts. Recognition of such atypical presentations is important for timely genetic counseling and screening of female carriers, as well as for the treatment of the patients, who may benefit from hematopoietic stem cell transplantation or gene therapy. Regarding *WAS* locus, variants located within the core promoter element c.−28 could also be candidate pathogenic variants. The mechanism of pathogenicity for variants in CRE is usually decreased transcription of otherwise normal gene products, resulting in a quantitative rather than qualitative defect^3^. Furthermore, some variants in CRE, may also lead to additional alternative splicing. Interestingly, variants in CRE that can also result in increased transcript products have been reported in other genetic diseases such as developmental disorders^3^, but not, to the best of our knowledge, for IEI. A limitation of the study is that we did not address the possible cell-specific impact of the variant^3^. Overall, the variant we report here further exemplifies that the implementation of bioinformatics tools for non-coding regions is warranted to systematically screen genetic data for variants in such regions in genes for IEI.

## Figures and Tables

**Figure –:**
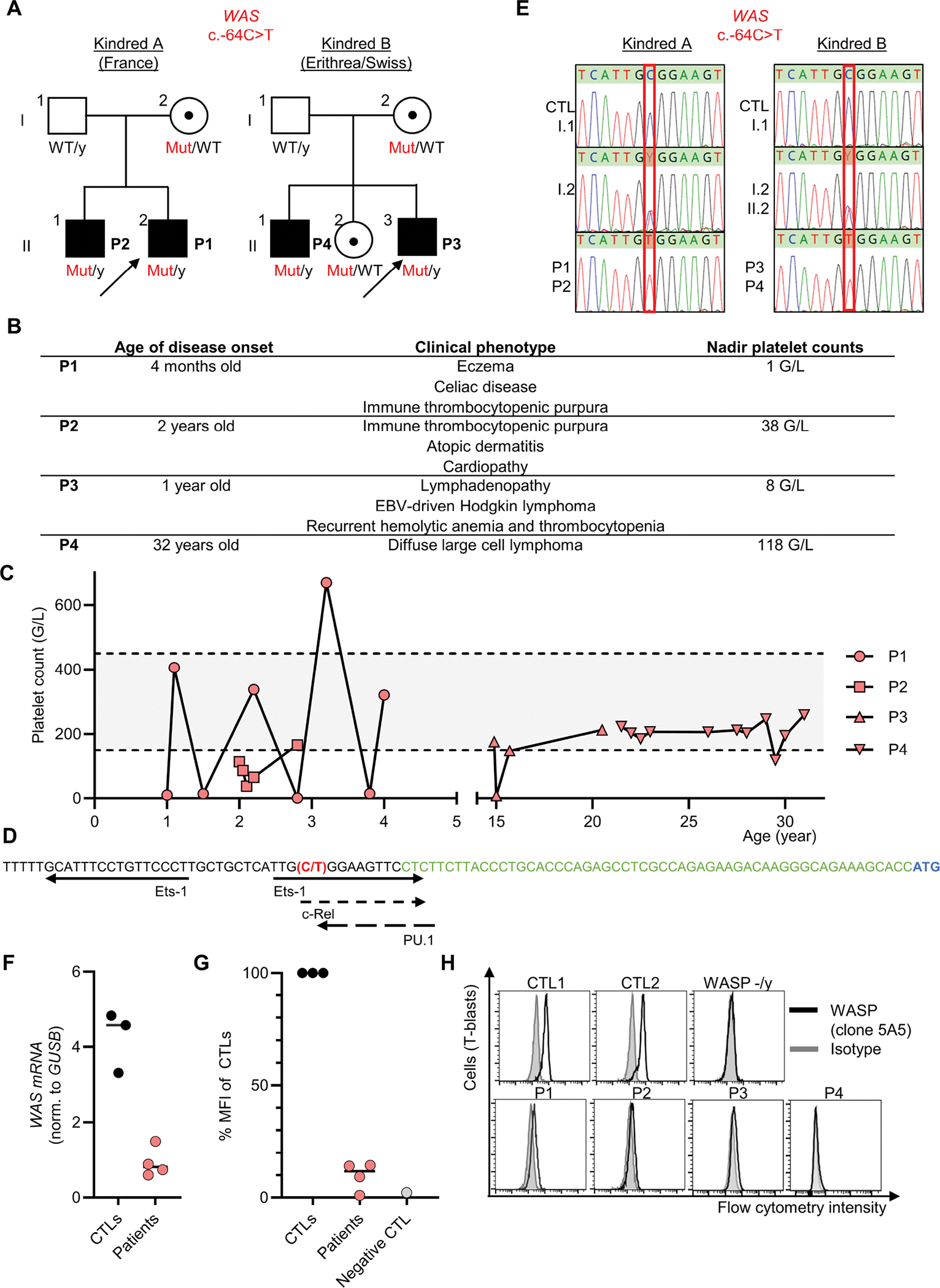
A single nucleotide promoter variant underlying Wiskott-Aldrich disease. **(A)** Pedigree tree. Male and female individuals are represented by squares and circles, respectively. Each generation is designated by a Roman numeral and each individual by an Arabic numeral. Individuals with immune dysregulation are shown as closed black symbols and the index case is indicated by an arrow. Black dots represent heterozygosity. Mut=mutated; WT=wild-type. **(B)** Summary of the phenotype of the four patients. **(C)** Counts of platelets in whole blood of the patients. Normal range is displayed in grey. **(D)** Scheme of *WAS* promoter adapted from Petrella *et al.1*^4^. Arrows indicate transcription factor binding sites identified by a combination of computational predictions and experimental validation. The c.−64C>T variant is displayed in red, and the 5’UTR and transcription start site of *WAS* in green and blue, respectively. **(E)** Electropherograms for the sequencing of representative *WAS* nucleotide sequences present in the hemizygous state in the four patients, in the heterozygous state in their mothers and sister, and in the homozygous wild-type state in control (CTL) and in their fathers. **(F)** *WAS* qPCR (norm. to *GUSB*) in T-activated cells from three controls and in the four patients**. (G)** Quantification of WASP protein expression derived from panel D. **(H)** Intracellular protein expression of WASp (clone 5A5) or isotype, in T-activated cells derived from two controls (CTL1 and CTL2), P1, P2, P3, and P4, and from a patient previously reported as deficient in WASP^1^.

## Data Availability

All data are either included in the manuscript or available upon request.
